# The effect of insurance status and parental education on glycemic control and cardiovascular disease risk profile in youth with Type 1 Diabetes

**DOI:** 10.1186/2251-6581-13-59

**Published:** 2014-05-22

**Authors:** Shideh Majidi, R Paul Wadwa, Franziska K Bishop, Georgeanna J Klingensmith, Marian Rewers, Kim McFann, David M Maahs

**Affiliations:** 1 Department of Pediatrics, Children’s Hospital Colorado, 13123 E. 16th Ave, Aurora, CO 80045, USA; 2 Barbara Davis Center for Childhood Diabetes, University of Colorado Denver, 1775 Aurora Ct, Aurora, CO 80045, USA

**Keywords:** Type 1 diabetes, Socioeconomic status, Parental education, Hemoglobin A1c, Cardiovascular disease

## Abstract

**Background:**

Adult studies have shown a correlation between low socioeconomic status and Type 1 Diabetes complications, but studies have not been done in children to examine the effect of socioeconomic status on risk for future complications. This study investigates the relationship between insurance status and parental education and both glycemic control and cardiovascular disease (CVD) risk factors in youth with type 1 diabetes.

**Methods:**

A cross-sectional study of 295 youth with established type 1 diabetes who underwent examination with fasting blood draw and reported insurance status and parental education.

**Results:**

Youth with type 1 diabetes and public insurance had higher hemoglobin A1c (HbA1c), body mass index, hs-CRP, and blood pressure (p < 0.05) than those with private insurance. Insulin regimen varied between insurance groups, and differences in HbA1c and CVD risk factors, except for diastolic blood pressure (DBP), were no longer evident after controlling for insulin regimen. Parental education was not associated with HbA1c or CVD risk factors.

**Conclusions:**

Youth with type 1 diabetes and public insurance have worse glycemic control and elevated CVD risk factors compared to those with private insurance, but this was no longer seen when insulin regimen was controlled for. Further research is needed to look at differences between those with public insurance and private insurance that contribute to differences in type 1 diabetes outcomes, and to identify modifiable risk factors in pediatric patients in order to focus earlier interventions to decrease and prevent future diabetes complications*.*

## Background

Cardiovascular disease (CVD) is the leading cause of mortality in patients with diabetes [1]. Vascular changes are seen in type 1 diabetes during adolescence [2,3] and lead to premature CVD in adults [4]. Intensive glycemic control decreases rates of vascular complications [5], emphasizing the importance of early intensive diabetes management and modifiable factors that affect glycemic control and CVD risk.

In adults, lower socioeconomic status predicts worse glycemic control as well as increased rate of complications [6,7] and mortality [8]. In the pediatric general population, children whose parents had less than a high school education had worse health compared to other children. In the United States, insurance status reflects one’s socioeconomic status, and those with public or no insurance are more likely to lack a source of care or get needed medical care [9]. In children with type 1 diabetes, lower socioeconomic status has been associated with worse glycemic control [10,11]. Specifically, underinsurance predicted increased incidence of severe hypoglycemia and diabetic ketoacidosis (DKA) [12,13]. Findings on the effect of parental education on glycemic control are inconclusive [14,15]. In studies showing an effect of parental education on glycemic control, it is often the father’s education level that is associated with glycemic control [15,16].

The goal of this study was to explore the association between parental education and insurance status and both glycemic control and cardiovascular risk factors. We hypothesized that those with lower parental education level or public insurance would have worse glycemic control and cardiovascular risk profiles.

## Methods

### Study design

As part of a prospective cohort study, families of youth with type 1 diabetes (n = 295) ages 12–19 years and with diabetes duration >5 years completed questionnaires and had fasting blood draws at a study visit between 2008 and 2010. This was a convenience sample of consecutive patients in a large academic pediatric diabetes clinic. Exclusion criteria included a history of abnormal cardiac anatomy or arrhythmia. This research study was reviewed and approved by the Colorado Multiple Institutional Review Board.

### Data collection

All families completed the insurance status portion of the questionnaire, asking whether they had insurance and if so to list their current insurance carrier. Public insurance includes Medicaid and Colorado Access, which includes Child Health Plan Plus (CHP+). Two hundred ninety-one subjects completed the parental education portion of the questionnaire which were listed as 0-20+, and separated for analysis similarly to SEARCH studies [17] with both parents’ education levels obtained and the highest value used for analysis (0–11 years for less than high school graduate, 12 for high school graduate, 13–15 for some college, and 16-20+ for college graduate or more). Race/ethnicity was self-reported using the 2000 U.S. Census definitions and categorized as non-Hispanic white (NHW), Hispanic or Other.

Subjects’ height, weight, and systolic and diastolic blood pressures (SBP, DBP) were obtained using standardized methods. Height, weight, and blood pressure were taken three times and the averages were calculated. Fasting laboratory measurements included: hemoglobin A1c (HbA1c), total cholesterol (TC), triglycerides (TG), HDL-c, calculated LDL-c via the Friedewald equation, and high sensitivity C-reactive protein (CRP). All measurements were carried out using standard methods in the University of Colorado Denver Clinical Research laboratory.

### Statistical analysis

Variables were checked for the distributional assumption of normality. HbA1c, TC, HDL-c, TG, and CRP were highly skewed; therefore, a natural log transformation was applied and transformed variables were used in analyses. ANCOVA with a Tukey-Kramer p-value adjustment was used to determine the effect of parental education and insurance status on glycemic control and cardiovascular risk factors after adjusting for age, sex, race/ethnicity category, and insulin regimen (defined as “pump” or “injection”). Estimates were reported as mean ± standard deviation (SD) for normally distributed variables and geometric mean and 95% confidence interval (CI) for non-normally distributed variables. Chi Square test of independence or Fisher’s Exact were used to test differences in gender distribution among categories. ANCOVA p < 0.05 was considered significant. Adjusted p-values are presented for pair-wise comparisons. ANCOVA p-values are presented in Table 1.

**Table 1:** Participant characteristics by insurance status

	**No insurance**	**Public insurance**	**Private insurance**	**P-value**
	**(N = 7)**	**(N = 36)**	**(N = 252)**	
Age (years)	14.6 ± 2.1	14.8 ± 2.2	15.5 ± 2.1	0.18
Sex (% Males)	43%	47%	52%	0.81
Duration of diabetes (years)	8.0 ± 3.0	8.6 ± 3.1	8.8 ± 3.0	0.91
Race/ethnicity (%NHW)	71%	53%	85%**	0.0001
Pumps	1 (16.7%)	11 (30.6%)	153 (61.2%)**	0.0002
BMI (%)	73 ± 27	79 ± 17	68 ± 23	0.07^
HbA1c (%)^#^	8.7 (7.5–10.1)	9.6 (9.1–10.1)	8.7 (8.5–8.9)	0.10^
HDL-c (mg/dL)^#^	51 (43–61)	50 (47–54)	50 (49–51)	0.78^
LDL-c (mg/dL)^#^	77 (60–100)	92 (84–100)	84 (81–87)	0.15^
Total Cholesterol (mg/dL)^#^	144 (123–168)	163 (153–173)	152 (148–156)	0.16^
Triglycerides (mg/dL)^#^	66 (52–85)	87 (75–101)	75 (70–79)	0.18^
CRP (mg/L)^#^	0.55 (0.30–1.02)	0.93 (0.60–1.45)	0.61 (0.52–0.71)	0.09^
SBP (mmHg)	118 ± 11	116 ± 11	113 ± 8	0.11^
DBP (mmHg)	70 ± 8	72 ± 7	68 ± 6**	0.01^

Note: **p < 0.01 for comparison between private insurance and public insurance.

^#^Geometric mean and 95% confidence interval.

^P-values are for ANCOVA with a Tukey-Kramer p-value adjustment (adjusting for age, sex, race/ethnicity category, and insulin regimen).

## Results

Comparison by insurance status showed that children with private insurance were more likely to be non-Hispanic white, have lower HbA1c, CRP, SBP, and DBP (p < 0.05). When insulin regimen was controlled for, only DBP remained significant (p = 0.01; Table 1). TC, TG, and LDL-c levels were also lower in children with private versus public insurance, but did not reach statistical significance. Insulin pump therapy was used more often in those with private insurance, compared to those with public insurance or no insurance (p = 0.0002).

In contrast, no difference in demographic characteristics (including race/ethnicity), glycemic control, or CVD risk factors were seen when comparing children with different parental education levels. However, insulin pump therapy was more likely to be used in those with college or graduate education (p = 0.0008).

## Discussion

This is the first study in children with type 1 diabetes, to our knowledge, to evaluate the association between markers of socioeconomic status and cardiovascular risk factors. This study found a worse profile of CVD risk factors in children with public versus private insurance, as well as worse glycemic control, but this association was no longer seen when insulin regimen was controlled for, except for diastolic blood pressure. Research in adults with type 1 diabetes indicates those with lower socioeconomic status have more diabetes complications [6,7], but this research is lacking in the pediatric population. One of the advantages of this study is the evaluation of a variety of risk factors for CVD in a pediatric population before the onset of significant disease. This allows the opportunity to identify CVD risk factors in at-risk populations.

This study showed a difference in insulin regimen between insurance types, and HbA1c and several cardiovascular disease risk factors were no longer significant after insulin regimen was controlled for. A recent meta-analysis looking at differences in HbA1c between children on continuous subcutaneous insulin infusion (CSII) versus multiple daily injections (MDI) showed no significant difference, although there was a trend toward better glycemic control in children on CSII and more data are needed on this topic [18]. Previous studies show those with a higher parental education level and those with private insurance are more likely to be on insulin pump therapy [19]. Within the institution where this study was conducted, there is no difference in access to diabetes clinic resources based on insurance status. Public insurance, such as Medicaid, covers pump therapy as well as private insurance does, but difference in pump use persists. For those with public insurance, other factors may restrict their access to resources and equal care. This study was not aimed at determining which factors in those with public or no insurance may affect their access to care. This is an important further step in research, to determine what the barriers are to those with public insurance obtaining access to resources and equal care that lead to the differences seen in this study.

Another limitation of this study is that certain variables that may also influence glycemic control and subsequent CVD status, such as frequency of self-monitored blood glucose, frequency of clinic visits, and other markers of patient support, were not investigated within the scope of this research. Further research is needed to address how these might be affected by insurance and socioeconomic status, and whether they are independent markers of subsequent cardiovascular disease. Such studies could identify variables which may be amenable to intervention strategies, and also highlight important disparities in access to health care.

Parental education did not have an effect on glycemic control or CVD risk prolife. Our questionnaire, as in the SEARCH study [17], did not identify the parent associated with each education level, which is a limitation in our study as analyses could not be done to determine effect based on paternal or maternal education level. Previous studies have shown an effect based on paternal, but not maternal education level [15,16] and not distinguishing between these two may have contributed to the lack of associations in this study.

In summary, this study showed that public insurance is associated with worse glycemic control and increased cardiovascular risk factors, but the association no longer remained after adjusting for insulin regimen. This may be a marker of financial difficulties in the family. Patients with public insurance are less likely to receive routine care [9]. In diabetes, this may translate to missing clinic appointments, although this has not been specifically investigated in type 1 diabetes youth with public or no insurance, including in this study. Parental employment status was not a component of our questionnaire, but could be evaluated in further studies to examine potential reasons behind the difference between patients with public and private insurance that lead to these discrepancies in outcomes. Further research is needed to look at discrepancies between those with public insurance and private insurance that may lead to differences in diabetes outcomes, and to identify modifiable risk factors in pediatric patients in order to focus earlier interventions to decrease and prevent future diabetes complications*.*

## Competing interests

The authors declare that they have no competing interests.

## Authors’ contributions

SM wrote this article. DM and PW are principal investigators for the study and reviewed/edited the article. KMF provided data analysis. FB is the study coordinator for research and reviewed/edited the article. GK and MR reviewed and edited the article. All authors read and approved the final manuscript.
